# Transcriptome profiling of wheat glumes in wild emmer, hulled landraces and modern cultivars

**DOI:** 10.1186/s12864-015-1996-0

**Published:** 2015-10-13

**Authors:** Hongda Zou, Raanan Tzarfati, Sariel Hübner, Tamar Krugman, Tzion Fahima, Shahal Abbo, Yehoshua Saranga, Abraham B. Korol

**Affiliations:** Department of Evolutionary and Environmental Biology, The Institute of Evolution, Faculty of Natural Sciences, University of Haifa, Haifa, 31905 Israel; Department of Botany, University of British Columbia, Vancouver, BC V6T 1Z4 Canada; Robert H. Smith Institute of Plant Sciences and Genetics in Agriculture, The Hebrew University of Jerusalem, Rehovot, 7610001 Israel

**Keywords:** Tetraploid wheat, Domestication, Glumes, RNA-seq, Differentially expressed genes

## Abstract

**Background:**

Wheat domestication is considered as one of the most important events in the development of human civilization. Wheat spikelets have undergone significant changes during evolution under domestication, resulting in soft glumes and larger kernels that are released easily upon threshing. Our main goal was to explore changes in transcriptome expression in glumes that accompanied wheat evolution under domestication.

**Methods:**

A total of six tetraploid wheat accessions were selected for transcriptome profiling based on their rachis brittleness and glumes toughness. RNA pools from glumes of the central spikelet at heading time were used to construct cDNA libraries for sequencing. The trimmed reads from each library were separately aligned to the reference sub-genomes A and B, which were extracted from wheat survey sequence. Differentially expression analysis and functional annotation were performed between wild and domesticated wheat, to identity candidate genes associated with evolution under domestication. Selected candidate genes were validated using real time PCR.

**Results:**

Transcriptome profiles of wild emmer wheat, wheat landraces, and wheat cultivars were compared using next generation sequencing (RNA-seq). We have found a total of 194,893 transcripts, of which 73,150 were shared between wild, landraces, and cultivars. From 781 differentially expressed genes (DEGs), 336 were down-regulated and 445 were up-regulated in the domesticated compared to wild wheat genotypes. Gene Ontology (GO) annotation assigned 293 DEGs (37.5 %) to GO term groups, of which 134 (17.1 %) were down-regulated and 159 (20.4 %) up-regulated in the domesticated wheat. Some of the down-regulated DEGs in domesticated wheat are related to the biosynthetic pathways that eventually define the mechanical strength of the glumes, such as cell wall, lignin, pectin and wax biosynthesis. The reduction in gene expression of such genes, may explain the softness of the glumes in the domesticated forms. In addition, we have identified genes involved in nutrient remobilization that may affect grain size and other agronomic traits evolved under domestication.

**Conclusions:**

The comparison of RNA-seq profiles between glumes of wheat groups differing in glumes toughness and rachis brittleness revealed a few DEGs that may be involved in glumes toughness and nutrient remobilization. These genes may be involved in processes of wheat improvement under domestication.

**Electronic supplementary material:**

The online version of this article (doi:10.1186/s12864-015-1996-0) contains supplementary material, which is available to authorized users.

## Background

Domestication of plants was a major event in the establishment of agriculture and human civilization. Wheat was among the first domesticated plant species and is considered as one of the most important crops in the world. Comparative studies of domesticated wheat with its wild progenitors lead to insights about the genetic basis of their adaptation which could be beneficial for future crop improvement. During domestication and subsequent crop improvement under domestication, numerous morphological and physiological characteristics of the wild progenitors were modified to meet human needs. The first and pristine domestication trait in cereals, non-brittle rachis, is related to the loss of kernel dispersal mechanisms. As a result, there was a transition from shattering hulled forms of wild einkorn wheat (*T. boeoticum* L., A^b^A^b^) and wild emmer wheat (*T. turgidum* L. ssp. *dicoccoides*, A^u^A^u^BB, also known as *T. dicoccoides*), to non-shattering hulled (as hard-threshing) forms in the diploid einkorn wheat (*T. monococcum* L., A^m^A^m^) and tetraploid emmer wheat (*T. turgidum* L. ssp. *dicoccum*, A^u^A^u^BB), respectively. Later on, during the evolution under domestication, a variety of changes have occurred, related to the glumes toughness, proportion of kernel weight in the whole spike weight, shape and colour, seed dormancy, disease and pest resistance, and high productivity in a wide range of environment [1].

The genome of tetraploid wheat originated about 0.5 million years ago from an interspecific hybridization event between the *T. urartu* (A^u^A^u^) and an unknown B genome ancestor presumably related to *Aegilops speltoides*. The genome of hexaploid wheat has resulted from a second inter-specific hybridization between domesticated tetraploid cultivated emmer *T. dicoccum* (A^u^A^u^BB) and *Ae. tauschii* (DD) followed by genome duplication ~9,000 years ago [2]. Durum wheat (*T. turgidum* L. ssp. *durum*) is the predominant form that was selected from emmer and has free-threshing grain. Thus, *T. dicoccoides* is the progenitor of both durum and bread wheat, and is central to wheat domestication evolution [3, 4].

The genetic basis of events involved in plant domestication and the nature of selection in domesticated crops have been subjected to intense molecular genetics and genomics studies over the past two decades [5, 6]. A large number of wheat domestication-related genes have been identified through quantitative trait locus (QTL) mapping [7–11], genome-wide association studies [12], and cloning [13, 14]. QTL mapping was one of the major approaches in genetic studies of plant domestication evolution and improvement, as well as in unravelling the agronomic potential of their wild progenitors. Most QTL analyses of wheat domestication and improvement focused on spike traits, including brittle rachis (preventing seed shattering) [8, 15] and glumes toughness (ease of threshing) [9, 16]. Many QTL studies have demonstrated that major key domestication traits are controlled by a relatively small proportion of the genome, implying that either pleiotropy or tight linkage among several loci may be an important attribute in the evolution of domesticated crops [8, 11, 17]. Nowadays, dense SNP genetic maps are available for the traditional QTL analysis of populations derived from crosses of domesticated plants with their wild progenitors [18] as well as for the genome-wide association studies [19, 20]. Comparison of QTL map locations with genome sequencing or genome-wide SNP scanning has also been used to identify candidate genomic regions involved in selection during domestication [21, 22]. Cavanagh et al. [6] developed a high-throughput array to integrate 9 K gene-associated SNPs in a worldwide sample of 2994 accessions of hexaploid wheat including landraces and modern cultivars to characterize the impact of crop improvement on genomic and geographic patterns of genetic diversity. The results showed that there are minor genetic differences between landraces and cultivars. In another study, a wheat genotyping array was developed with about 90 K gene-associated SNPs, which is an excellent resource for fine-scale genetic dissection of domestication related traits [23].

Additional attempts to illuminate the domestication process by using functional genomics included expressed sequence tag (EST) sequencing, microarray and more recently, RNA-seq technologies. Ergen and Budak constructed six subtractive cDNA libraries and sequenced over 13,000 ESTs using wild emmer wheat accessions and modern wheat in order to analyse the expression profile of drought related genes [24]. The first microarray comparison between developing spikes of tetraploid wild (*T. dicoccoides*) and domesticated wheat (*T. dicoccum* and *T. durum*) at the stage of one week after pollination, identified 38 and 24 differentially up- or down-regulated genes, respectively, out of 2493 cDNA clones on the array [25]. Most of the genes that were found to be up-regulated in the domesticated wheat were related to carbon metabolism, such as *Rubisco large* and *small subunits* and the *sucrose synthase*. Among down-regulated genes in domesticated wheat the authors noted storage protein genes and genes associated with abiotic and biotic stress responses. Although comprehensive studies using the microarray had achieved a better understanding of the wheat genome expression [26, 27], the microarray technology has some limitations compared to RNA-seq. Microarray analysis relies on hybridization between probes and targets. Most microarray studies are based on commercial arrays such as the Wheat Genome Array (Affymetrix), where target transcripts were designed using EST libraries of cultivated wheat. Nevertheless, since there is high sequence similarity between wild and cultivated wheat, it was also successfully used for expression studies of wild emmer [28–30]. Nowadays, the advanced technology of Next Generation Sequencing (NGS), enabling to sequence the whole transcriptome (RNA-seq), was proved as an excellent approach to study changes in domestication related genes and expression networks underlying plant domestication and crop improvement [31–33]. NGS has remarkable advantages over the microarray in the detection of novel transcripts, allele-specific expression and splice junctions [34]. Hence, RNA-seq can expand our view and provide new insights into plant domestication evolution at the genomics level.

Wheat glumes are an important part of the spikelet, which is the dispersal unit of the plant. Genes involved in development and structure of the glumes and spikes are interesting from both theoretical and practical aspects [35]. The glumes are the closest vegetative tissue to the grain. As part of their role in reproduction, the glumes serve as a ‘defense line' for the kernels, and act on nutrient allocation and photo-assimilates conversation destined for the developing kernels [36]. The glumes composition and structure can greatly impact plants performance and their interaction with environment. Recently, it was suggested that glumes can serve as a photosynthetically active sinks adjusting for the changing metabolism demand of the kernels [37]. Glumes can also maintain their metabolic activity longer than other vegetative organs and influence the final yield and nitrogen cycling [38]. Moreover, there is indication that glume phenotype has a possible correlation with some beneficial agronomic traits [39]. Genes affecting glumes, like *Q* in wheat and *tga1* in maize, were involved in key steps of domestication and are related to diverse biological functions, implying significant roles of the glumes [13, 40]. As noted above, wheat glumes have undergone significant changes along evolution under domestication. The main outcome of this process was the reduction in glumes toughness and the increase of the kernels weight proportion in the total spike weight (SpHI, spike harvest index) [16].

In the current study, we explored the evolutionary changes of the tetraploid wheat transcriptome by comparative RNA-seq analysis of three dissimilar genotypic groups, wild emmer wheat, tetraploid landraces and modern *T. durum* cultivars, representing three different time points in wheat domestication. We have identified large differences in gene expression between the wild and domesticated wheat. Among the differentially expressed genes, we identified genes that may be involved in glumes toughness and threshability, nutrient remobilization and the proportion of kernels in the whole spike weight and other agronomic traits evolved under domestication.

## Methods

### Plant material

A total of six tetraploid wheat accessions were selected for transcriptome profiling based on their rachis brittleness and glumes toughness [16]. These included: (1) two wild emmer wheat *T. dicoccoides* (accessions Y12-3 and A24-39) characterized by brittle rachis and tough (hulled) glumes; (2) traditional landraces including *T. dicoccum* (G581) and *T. ispahanicum* (G805) characterized by non-brittle rachis and tough glumes; and (3) two modern cultivars of *T. durum* (‘Inbar’ and ‘Svevo’), characterized by non-brittle rachis and soft glumes (Table 1).

**Table 1:** Wild, landrace and cultivar tetraploid wheat genotypes used in the study

Group	Species	Accessions	Rachis and glumes characterization
Wild	*T. turgidum* L. *subsp. dicoccoides*	Y12- 3	Brittle rachis, hard to thresh
		A24-39	
Landrace	*T. turgidum* L. *subsp. dicoccum*	G 805	Non-brittle rachis, hard to thresh
	*T. ispahanicum* Heslot	G 581	
Cultivar	*T. turgidum subsp. durum* (Destf.)	Inbar	Non-brittle rachis, soft glumes
		Svevo	

Plants were grown in three biological replicates as described in [16]. Glumes of the central spikelet of each genotype were sampled at its heading time (when the spike was fully emerged). Each accession was sampled independently 1 h after sunrise. Glumes were collected, placed immediately in Eppendorf tubes with RNAlater (Qiagen, Hilden, Germany), and stored at −20 °C for RNA extraction.

### RNA extraction and sequencing

RNA was extracted from glumes using the Plant Mini Kit including a digestion step with DNase I (Qiagen, Standford, CA, USA) for removal of DNA traces. High quality RNA was confirmed using Bioanalyzer 2100 with RNA 6000 Nano Labchips (Agilent, Santa Clara, CA, USA). RNA samples were pooled to three groups in accordance with their level of domestication, i.e., wild, landraces and cultivars. As the main objective of this study was to identify transcription differences along domestication “gradient”, pooling samples should give higher credence to representative genes of groups rather than genotypes. Each of the pools contained 1 μg RNA of the two accessions (Table 1). For each RNA pool, two independent biological replicates (i.e., six pools) were used to construct RNA-seq libraries, and a third replicate was reserved for QPCR validation. The cDNA libraries were constructed using NEBNext Ultra Directional RNA Prep Kit (New England Biolabs, MA), following the manufacturer’s instructions. After verifying the quality of the libraries indexed with six-nucleotide barcodes, sequencing was performed on the Illumina Hiseq2000 machine using multiplexing for generating 2 × 101 bp paired end reads. Sequencing was carried out at the Technion Genome Center (Haifa, Israel).

### Data processing, mapping and SNPs discovery

A tetraploid reference genome was prepared *in silico* by extracting sequences assigned to the A and B genomes from the chromosome survey sequencing (CSS) data of the IWGSC (International Wheat Genome Sequencing Consortium, http://www.wheatgenome.org) [41]. Sequences from each RNA-Seq pool were cleaned and trimmed by removing adaptor sequences and low-quality reads using Trimmomatic software (version 0.32) [42] with the following parameters: phred64, LEADING: 3, TRAILING: 3, SLIDINGWINDOW:4:20, MINLEN:40 (phred quality scores Q ≥ 20, read length ≥ 40). Each cleaned library was aligned to each of the tetraploid reference subgenomes separately, using the Subjunc aligner in Subread package (version 1.43) [43] with the following parameters: -d 0, -D 1000, -u, -H, -I 16, -S fr. The -u option was used to report uniquely mapped reads only, whereas -H option was used to breaks ties using Hamming distance when there was more than one best mapping location for a read, which would give the most accurate mapping results with little or no cost to the mapping percentage.

Because it is not feasible yet to index a large genome (more than 4 Gbp) by *Subread*, we had to split the wheat reference genome AABB into sub-genome A and sub-genome B, and then combine the alignment results using the following method. After alignment, the sum of mapping quality scores (MQS) for each mapped read was used to determine to which sub-genome (A or B) the read should be assigned. For accurate alignment, the read pairs had priority over singletons (when only one read of a pair was mapped) and uniquely mapped reads have priority over ambiguously mapped reads. When the same read was mapped to the two genomes, the genome with the higher MQS was accepted and the other one was discarded. The read that had the same alignment score in the two genomes was discarded by the custom script (such reads comprised a very low percentage). This may be an applicative methodology whenever the genome size exceeds the tools limitation that can help us to further characterize homoeolog-specific reads.

Genotype calling was carried out with the alignment files using SAMtools/BCFtools (version 0.1.19, http://samtools.sourceforge.net) with default parameters. All SNPs with maximum read depth less than 100 were kept for subsequent analysis. The relationship between the mapping ratios and genetic distance from reads to reference genome were examined by Pearson correlation.

### Differential gene expression analysis

We further used featureCounts [44] in the Subread package to quantify the level of expression for each gene based on the associated gtf (Gene Transfer Format) file provided with the survey sequence. In order to reveal differentially expressed genes (DEGs) in domesticated vs. wild accessions, we considered the common part of two subsets: DEGs between cultivated vs. wild and DEGs between landrace vs. wild. DEGs in each of these two comparisons were identified using DESeq software (version 1.6.1) [45] at selection cutoff log2Foldchange ≥ 1 and 10 % FDR (False Discovery Rate), implying that *p*-values were adjusted for multiple testing based on Benjamini-Hochberg approach at a level below 0.1.

### Functional analysis of differentially expressed genes

Gene Ontology (GO) terms were searched with Blast2GO [46]. First, we extracted the sequences of the DEGs from reference genomes and gtf files with a custom script. Then the sequences of DEGs were compared to the NCBI nr (non-redundant) database using blastx with a cutoff e-value less than 1e-5 [47]. The blastx output, generated in xml format, was used for Blas2GO analysis to annotate the DEGs. GO functional classification for DEGs was performed using the WEGO software [48].

### Unmapped reads processing, *de novo* assembly, differential gene expression analysis and functional annotation

Reads that failed in the alignment procedure were extracted from alignment files using SAMtools (version 0.1.19); and assembled *de novo* in Trinity (version 2.06) [49] with default parameters. We further aligned the raw unmapped reads of each group to the assembled contigs with Bowtie [50] and estimated genes abundance using RSEM [51]. Because most of the reads identified as unmapped were essentially ambiguously mapped, assembled transcripts with high identity (>70 %) to the IWGSC reference genome found by blastn were discarded. The remaining transcripts were annotated with Blast2GO as mentioned above and used to identify differentially expressed transcripts (DETs) among the three groups using edgeR with default parameters [52].

### Quantitative reverse transcription PCR (QRT-PCR)

A few transcripts were selected for validation of RNA-seq results by QRT-PCR as described in [29, 30]. Total RNA (1 μg) from relative samples were used to generate complementary DNA (cDNA) templates using the qScript cDNA synthesis kit (Quanta BioSciences, Gaithersburg, MD, USA). Gene-specific primers and SYBR Green PCR master mix (Quanta BioSciences, Gaithersburg, MD, USA) were used for QPCR on StepOne System (Applied Biosystems, Darmstadt, Germany), according to the manufacturer’s instructions. Gene-specific primers were designed by Primer3 (http://primer3.ut.ee/primer3) (Table 2). PCRs included 2.5 μL of 1^st^-strand cDNA (1:4 diluted cDNA), 7.5 μL of SYBR Mix and 300 nM of each primer in a final volume of 10 μL. The primers were designed in non-polymorphic regions across accessions. We tested the efficiency of the primers using four serial dilutions (of 1:4) for each of the wheat groups. The specificity of each primer pair was monitored by heat dissociation curve analysis of the amplicon as a final step of the PCR. For *COBRA*, *CesA-1,* and *CCR* genes we used cDNA samples of three biological replicates per accession as a template for QPCR. For the other six genes, *FLA*, *FST*, *CesA-2*, *6-SFT*, *LAC* and *LAC16*, we used the cDNA pools similarly to the RNA-seq pools rather than individual cDNA (due to limited RNA with two to three technical replicates for each biological sample). The endogenous gene *Ubiquitin* was used to normalise variations within well-to-well and across plates with forward and reverse primers: 5’ - TTGACAACGTGAAGGCGAAG - 3’ and 5’ - GGCAAAG ATGAGACGCTGCT - 3’ respectively. Quantification of gene expression was carried out by the relative quantification method (2^-∆∆CT^ method) [53] implemented in StepOne software v2.3. Since efficiencies of the several primers were not identical in the three wheat group, we corrected the relative quantification results according to the efficiency of each of the genes in each of the groups by StepOne software v2.3, which is based on: Efficiency = 10 ^(−1/slope)^ for each primer and RQ = E_target_^- (Ct treatment - Ct control)^/E_ndogenous_^- (Ct treatment - Ct control)^.

**Table 2:** Primers for QRT-PCR

Gene	Gene ID	Forward (5’ – 3’)	Reverse (5’ – 3’)
*CCR*	Ta1alLoc003924.2	TGCCGTGAGAAGAAGGTGAT	CTTCTCTGCCATCGTCTTGC
*COBRA*	Ta5asLoc003744.1	CGTCGCCGTTGAAGTAGATCT	TCATCGCAAGGATGATAGAAC
*CesA-1*	Ta5alLoc000723.1	GTCAAGCAAGAACAGCAT	ACCAGCTACCCACAAGAGCAA
*FLA*	Ta3bLoc003710.1	CAGTACCCGCTCAACGTCAC	CCGGTGTAGAGCGTGTTGTC
*FST*	Ta3bLoc056384.1	TGAACATGAGCAAGCTGGAG	CCATTCTGTCTCCCATGTCC
*LAC16*	Ta4asLoc013789.1	GTCCGATCTACCCGTCTGTT	GTGTGTTTATGCAAACCAAAGG
*LAC*	Ta4blLoc021918.2	GCAGAAGGTGACACGGCTAT	GCCTTCCCTTGTGACGATT
*CesA-2*	Ta5alLoc000723.1	ATCAGGCTTTGATTTCAGCAA	TGGATGTCATGTCAAGCAAGA
*6-SFT*	Ta6bsLoc005412.1	AGCTGTCAGTGAGGGTGCTT	CGTTGGGTACACTCGTGATG

## Results

### Assembly of reads into transcripts of the A and B genomes

Sequencing of mRNA from glume tissue of wild, landrace and modern tetraploid wheat pools generated 147.4 million pair-end reads of 101 bp length. The number of reads from each pool ranged from 17.7 to 29.6 million (Table 3). After removal of ambiguous nucleotides, low-quality sequences (phred quality scores <20), and adapter sequences, a total of 139.6 million cleaned reads were used to quantify the level of expression by mapping to the tetraploid reference sequences extracted from the wheat chromosome survey sequencing (CSS) data. Without the -u flag in Subjunc, the mapping ratios of sequences corresponding to the A and B genomes of wheat ranged between 75.1 and 79.6 % in the A genome and from 77.5 to 82.2 % in the B genome. With the -u flag, these ratios ranged from 56.5 to 64.6 % in the A genome and from 56.9 to 65.2 % in the B genome. After assigning each read to the corresponding genome based on mapping quality score, the mapping ratio over all remaining reads reached a range of 84.4–87.4 % without -u option and 71.3–83.1 % with -u option. Therefore, we used the results obtained by using the -u flag to maintain a conservative approach for downstream analysis.

**Table 3:** Summary of samples and RNA-seq data

Groups	Total reads	Clean reads	Sub-genome	Mapped reads	Mapping ratio (%)	Total mapping ratio (%)	Mapped reads -u	Mapping ratio (%) -u	Total mapping ratio (%) -u
Wild-1	23,271,884	22,088,185	A	17,586,172	79.6	86.5	13,102,552	59.3	74.6
			B	17,813,886	80.6		13,140,929	59.5	
Wild-2	27,577,198	26,160,151	A	20,700,875	79.1	85.9	15,242,936	58.3	73.7
			B	21,023,950	80.4		15,242,734	58.3	
Cultivar-1	17,740,180	16,773,599	A	13,527,727	80.6	87.4	9,474,682	56.5	71.3
			B	13,783,371	82.2		9,535,891	56.9	
Cultivar-2	29,551,798	27,977,895	A	22,256,822	79.6	86.6	16,395,908	58.6	74.3
			B	22,663,043	81.0		16,290,987	58.2	
Landrace-1	23,082,003	21,898,663	A	16,563,967	75.6	85.3	14,141,135	64.6	83.1
			B	16,971,148	77.5		14,275,683	65.2	
Landrace-2	26,163,934	24,724,657	A	18,580,284	75.1	84.4	15,687,853	63.5	81.9
			B	19,196,718	77.6		15,843,276	64.1	
Total	147,386,997	139,623,150							

To test whether genetic similarity between each pool and the reference sequences has an effect on mapping ratio we compared the genetic distance calculated from variant called among pools and mapping ratio. No correlation was found between genetic similarity and mapping ratio either with (r = 0.12, *p* = 0.701) or without (r = 0.41, *p* = 0.182) the -u flag (Additional file 1: Figure S1). These results further support our analytical approach and corroborate the downstream expression results.

### Differentially expressed genes (DEGs) between domesticated and wild wheat

**Fig. 1:**
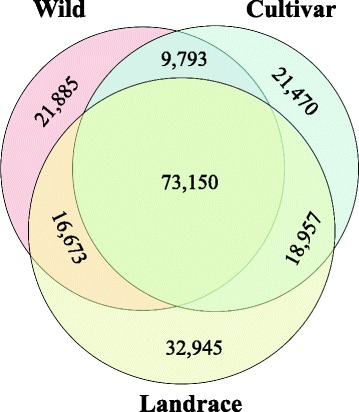
Proportional Venn diagram of transcripts among wild wheat genotypes, cultivars and landraces

**Fig. 2:**
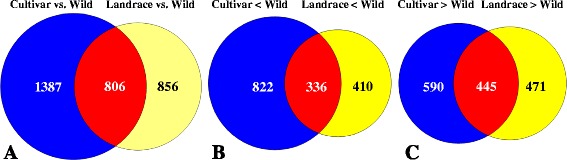
Proportional Venn diagrams of DEGs in domesticated compared to wild wheat. **a** Total DEGs. **b** DEGs down-regulated in domesticated wheat. **c** DEGs up-regulated in domesticated wheat

**Fig. 3:**
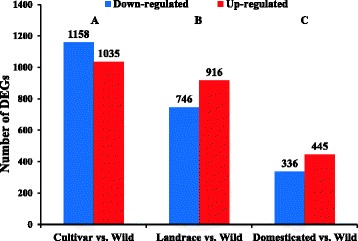
Histograms of DEGs in cultivar, landrace and domesticated compared to wild wheat. **a** DEGs in cultivar genotypes. **b** DEGs in landraces. **c** DEGs in domesticated wheat

**Fig. 4:**
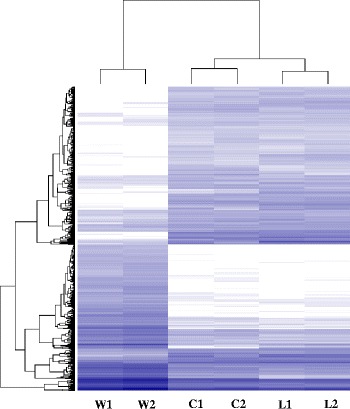
Heat map of DEGs in glumes of domesticated vs. wild wheat. The heat map represents the genes expression level of the 781 significant DEGs between wild and domesticated wheat (cultivar plus landrace) from all the six groups (log2Foldchange ≥ 1 and FDR ≤ 0.1). *Blue color* indicates gene expression level. Wild is abbreviated to W, Cultivar is abbreviated to C, and Landrace is abbreviated to L

A total of 194,893 transcripts were found expressed in wild, landrace and cultivar pools, out of which 73,150 transcripts were commonly expressed in the three groups (Fig. 1). Differential expression was first compared between the wild wheat and each of the two groups of domesticated wheat (landraces or modern cultivars) and then between wild and domesticated (landrace + modern cultivars). We found a higher number of DEGs (2193 DEGs) in the comparison of modern vs. wild wheat than in the comparison between landraces vs. wild wheat (1662 DEGs). In the modern cultivar vs. wild, 1035 DEGs were down-regulated and 1158 DEGs were up-regulated in modern cultivars as compared to the wild progenitor (Additional file 2: Table S1). A total of 746 DEGs were down-regulated and 916 DEGs were up-regulated in landraces as compared with the wild progenitor (Additional file 3: Table S2). The comparison between the domesticated (landraces + modern cultivars) vs. wild accessions identified only 781 DEGs, of which 445 genes had higher expression in the domesticated and 336 DEGs had higher expression in the wild wheat (Figs. 2 and 3; Additional file 4: Table S3). A heat-map of 781 significant DEGs between wild and domesticated pools was created using DESeq (Fig. 4), demonstrating clustering that distinguished between the domesticated (landraces and modern cultivars) and wild pools.

### Functional analysis of DEGs between wild and domesticated wheat

**Fig. 5:**
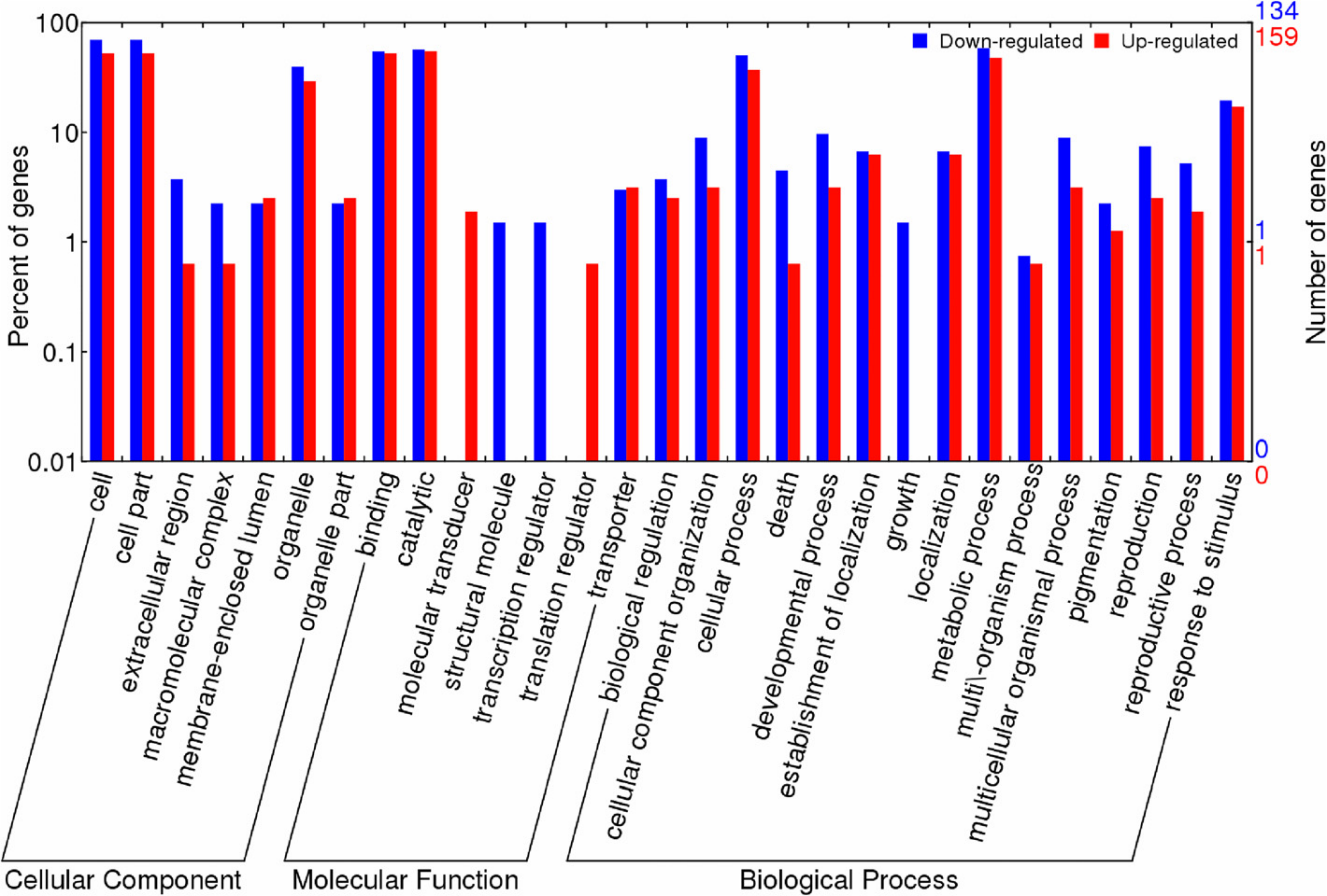
Comparison of Gene Ontology classifications of DEGs in domesticated vs. wild wheat. *Blue color* indicates down-regulated DEGs in domesticated compared to wild wheat, *red color* indicates up-regulated DEGs in domesticated compared to wild wheat. All of DEGs are categorized into 29 functional groups based on GO classification

In order to investigate transcriptome changes in glumes evolution under domestication, we assessed the expression patterns of the DEGs in domesticated (landraces + cultivars) vs. wild wheat (Additional file 4: Table S3). To annotate the DEGs in wild and domesticated groups, sequences were searched against the NCBI non-redundant (nr) protein database by blastx using a cut-off e-value of 10^−5^. GO terms were subsequently assigned to DEGs based on the blastx results. Out of 781 DEGs, only 293 DEGs (37.5 %) were assigned to GO-term groups, including 134 (17.1 %) DEGs down-regulated and 159 (20.4 %) DEGs up-regulated in the domesticated wheats compared to the wild accessions (Fig. 5). The DEGs were categorized into 29 groups based on GO annotation. The categories ‘cell, cell parts and organelles’, ‘binding and catalytic’ and ‘cellular process and metabolic process’ showed highest numbers of GO terms for the ‘cellular component’, ‘molecular functionality’ and ‘biological process’ categories, respectively. Interestingly, structural molecule and transcriptional regulator (in ‘molecular’ GO category) and growth (in ‘biological process’ GO category) were found only among the DEGs down-regulated in the domesticated genotypes compared to wild wheats. Molecular transducer and transition regulator in the ‘molecular function’ GO category were found only in DEGs up-regulated in the domesticated compared to wild wheats.

Based on gene annotation, we selected DEGs that could be regarded as candidate genes for domestication process in two possible directions of evolutionary changes (i.e., up- or down-regulated in the domesticated wheat compared to its progenitor). However, we selected the candidate genes based on uni-direction expression difference (either up- or down-regulated in the domesticated accessions). For example, three cellulose synthase genes were found only in the down-regulated DEGs in the domesticated wheat, whereas three amino acid permease genes were found only among the up-regulated DEGs in the domesticated wheat. A total of 22 DEGs were down-regulated in domesticated compared to wild wheat (Table 4). Using the available gene annotation [41], we found that many of these DEGs are related to cell wall organization or biogenesis; phenylpropanoid metabolism; and carbohydrate metabolism and transportation. For example, genes encoding for cellulose synthase (*CesA*), fasciclin-like arabinogalactan (*FLA*), trichome birefringence-like (*TBL*), fiber protein, pectin lyase-like protein and pectin acetylesterase family protein, and CER1 are related to cell wall organization and biogenesis. Genes encoding for phenylalanine ammonia lyase (*PAL*), cinnamoyl CoA reductase (*CCR*), flavonol 4-sulfotransferase (*FST*) and 4-coumarate:CoA ligase (*4CL*) are involved in phenylpropanoid metabolism and lignin biosynthesis; while genes encoding for sucrose synthase 2 (*SUS2*) and sucrose:fructan-6-fructosyltransferase (*6*-*SFT*) are responsible for carbohydrate metabolism and transportation. We further identified transcription factor genes down-regulated in domesticated wheat, such as genes encoding plant-specific transcriptional regulator NAC domain protein. Notably, *COBRA* genes have shown significant down-regulation in modern cultivar compared to wild wheat (see Table S1 and Discussion).

**Table 4:** DEGs down-regulated in glumes of domesticated wheat compared to wild progenitor

id	baseMean	baseMean	baseMean	log2Fold	Padj	log2Fold	Padj	Putative annotation
	(Wild)	(Cultiviar)	(Landrace)	Change(C/W)	(C/W)	Change(L/W)	(L/W)	
Ta7alLoc001275.1	1093.35	416.13	388.49	−1.39	7.96E-03	−1.49	3.19E-03	4-coumarate:CoA ligase
Ta1blLoc007155.1	5137.83	1403.42	2048.36	−1.87	1.00E-05	−1.33	6.17E-03	Cellulose synthase
Ta5alLoc000723.1	1933.05	441.04	647.98	−2.13	7.97E-07	−1.58	6.55E-04	Cellulose synthase
Ta3bLoc028980.1	4678.25	1223.81	1929.83	−1.93	3.84E-04	−1.28	8.50E-02	Cellulose synthase
Ta4alLoc006547.1	177.41	0.00	0.00	-Inf	2.47E-15	-Inf	1.30E-17	CER1 protein
Ta4alLoc026069.1	550.15	0.00	0.00	-Inf	8.29E-26	-Inf	4.65E-29	CER1 protein
Ta1alLoc003924.2	40.98	1.82	0.39	−4.49	5.99E-02	−6.71	1.80E-03	Cinnamoyl CoA reductase
Ta3bLoc003710.1	5610.65	1515.59	1849.21	−1.89	0.0001	−1.60	0.0016	Fasciclin-like arabinogalactan protein 7
Ta3bLoc056384.1	25.40	0.00	0.00	-Inf	6.75E-02	-Inf	3.53E-02	Flavonol 4-sulfotransferase
Ta4alLoc006913.1	299.00	4.85	7.32	−5.95	1.64E-16	−5.35	1.08E-16	Flavonol 4-sulfotransferase
Ta5blLoc013288.1	944.48	181.67	269.42	−2.38	1.04E-07	−1.81	1.66E-04	NAC domain-containing protein 18
Ta6blLoc001596.1	1507.23	377.78	671.34	−2.00	8.20E-06	−1.17	5.04E-02	Pectin lyase-like protein
Ta3bLoc036242.1	251.80	34.64	32.65	−2.86	1.16E-03	−2.95	1.53E-04	Pectinacetylesterase family protein
Ta3bLoc019897.1	219.61	36.13	30.72	−2.60	1.40E-02	−2.84	4.83E-03	Pectinacetylesterase family protein
Ta2blLoc014498.1	719.00	118.04	306.22	−2.61	4.62E-08	−1.23	6.04E-02	Phenylalanine ammonia-lyase
Ta3bLoc024051.1	342.10	34.22	106.23	−3.32	4.41E-09	−1.69	1.19E-02	Phenylalanine ammonia-lyase
Ta7asLoc021287.1	3214.99	932.09	1145.19	−1.79	3.10E-02	−1.49	8.21E-02	Sucrose synthase 2, putative, expressed
Ta6bsLoc005412.1	839.45	149.96	376.09	−2.48	3.87E-07	−1.16	8.73E-02	Sucrose:fructan-6-fructosyltransferase
Ta4alLoc019947.1	168.96	45.07	38.20	−1.91	1.72E-02	−2.14	5.10E-03	Fiber protein Fb34
Ta7bsLoc005648.1	161.29	39.24	43.95	−2.04	2.28E-02	−1.88	3.37E-02	TRICHOME BIREFRIGENE like 22
Ta4blLoc021918.2	235.61	19.18	49.36	−3.62	4.92E-08	−2.26	8.61E-04	Laccase
Ta4asLoc013789.1	939.60	192.99	304.20	−2.28	5.70E-07	−1.63	1.16E-03	laccase 16 LENGTH=523

In contrast to above down-regulated genes under domestication, 17 DEGs were found to be significantly up-regulated in domesticated wheat compared to the wild progenitor (Table 5). The most abundant groups of up-regulated DEGs in the domesticated pools included *3-ketoacyl-CoA synthase* (*KCS*) gene and *Chalcone synthase* (*CHS*) gene. Besides that, we also found *amino acid permease* (*AAP*) gene and *silicon transporter* (*SIT*) gene were up-regulated in domesticated wheat.

**Table 5:** DEGs highly up-regulated in glumes of domesticated wheat compared to wild progenitor

id	baseMean	baseMean	baseMean	log2Fold	Padj	log2Fold	Padj	Putative annotation
	(Wild)	(Cultiviar)	(Landrace)	Change(C/W)	(C/W)	Change(L/W)	(L/W)	
Ta7asLoc021951.1	42.47	1189.91	717.98	4.81	1.80E-04	4.08	2.50E-03	3-ketoacyl- synthase 12-like
Ta7bsLoc002749.1	2.79	306.18	109.26	6.78	5.12E-05	5.29	4.28E-03	3-ketoacyl- synthase 12-like
Ta6asLoc018551.1	0.00	53.86	18.54	Inf	4.91E-04	Inf	2.85E-02	3-ketoacyl-CoA synthase
Ta7asLoc001384.1	20.78	591.48	521.17	4.83	1.59E-02	4.65	2.39E-02	3-ketoacyl-CoA synthase
Ta7bsLoc001848.1	14.15	363.40	330.26	4.68	5.37E-02	4.54	8.58E-02	3-ketoacyl-CoA synthase
Ta7bsLoc002750.1	14.74	443.97	186.17	4.91	3.14E-03	3.66	7.64E-02	3-ketoacyl-CoA synthase
Ta7bsLoc005172.1	0.46	89.24	55.43	7.59	1.12E-02	6.90	3.76E-02	3-ketoacyl-CoA synthase
Ta7bsLoc011512.1	67.99	919.81	753.44	3.76	3.74E-03	3.47	1.19E-02	3-ketoacyl-CoA synthase
Ta6bsLoc008917.1	0.50	195.60	217.19	8.62	4.50E-10	8.77	3.18E-10	Chalcone synthase
Ta2bsLoc009111.1	0.46	1663.14	453.70	11.81	2.19E-16	9.93	5.54E-12	Chalcone synthase 8, putative
Ta3bLoc000987.1	0.93	1583.66	2159.97	10.74	4.24E-22	11.18	2.26E-23	Chalcone synthase 8, putative
Ta4bsLoc019947.2	0.00	51.71	97.56	Inf	8.90E-04	Inf	1.74E-04	Chalcone synthase 8, putative
Ta6bsLoc002330.1	0.00	306.31	386.67	Inf	3.33E-19	Inf	2.60E-20	Chalcone synthase 8, putative
Ta2alLoc009166.1	71.52	237.64	320.20	1.73	0.0077	2.16	6.86E-05	Amino acid permease 6
Ta2alLoc010251.2	11.86	139.49	88.59	3.56	0.00	2.90	6.08E-03	Amino acid permease-like protein
Ta5asLoc003267.1	28.62	147.37	107.71	2.36	0.00	1.91	2.31E-02	Amino acid permease
Ta1alLoc016727.3	36.90	268.40	132.25	2.86	2.30E-07	1.84	1.04E-02	Silicon transporter

### Unmapped reads processing, *de novo* assembly, differentially expression analysis and functional annotation

The unmapped reads extracted from six libraries were pooled together and *de novo* assembled using Trinity to generate a set of transcrips absent from the reference genome. From the unmapped reads, 64,316 contigs were assembled with length ranging from 224 bp to 24,492 bp and N50 of 494 bp. After removing transcripts that had high identity (>70 %) to the IWGSC reference genome, 7264 contigs ranging between 224 bp and 4296 bp were kept. These contigs are considered as novel transcripts. A total of 2761 novel transcripts had significant hit in searches against the nr database using blastx with cutoff 1e-5. GO analysis was conducted by Blast2go and GO terms were assigned to 1622 transcripts (Additional file 5: Figure S2). Differentially expressed transcripts were validated based on the protocol for downstream analyses of *de novo* assemble using Trinity (see section Methods). We found 110 DETs in modern vs. wild wheat, out of which 67 were down-regulated and 43 were up-regulated in modern cultivars as compared to the wild progenitor. We also found 111 DETs in landrace vs. wild wheat, out of which 68 were down-regulated and 43 up-regulated in landrace as compared to the wild progenitor. The comparison between the domesticated vs. wild accessions detected only 59 DETs, of which 52 had higher expression in the domesticated and 7 had higher expression in the wild wheat. It should be noticed that the overwhelming majority of these DETs have no known function and missing information about their sub-genome location (Additional file 6: Table S4).

### Quantitative reverse transcription PCR (QRT-PCR)

**Fig. 6:**
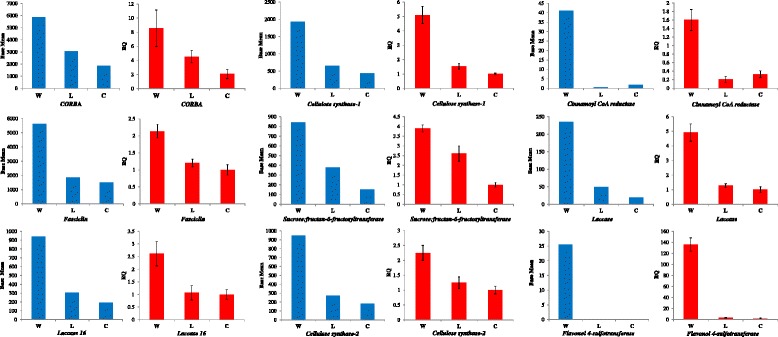
QRT-PCR validation of RNA-seq results for DEGs

To validate the RNA-seq results, QPCR was performed for nine selected DEGs that appeared to have higher expression in the glumes of the wild accessions. Moreover, based on gene annotation, these genes can be considered as interesting candidates for glumes evolution under domestication. PCR products of these genes’ primers amplified for each of the wheat groups (wild, landraces and cultivars) were specifically indicated by the single-peak melting curves. Due to the limited RNA, we tested the efficiency of the primers of gene *FLA*, *FST*, *CesA-2*, *6-SFT*, *LAC* and *LAC16*, using four serial dilutions (of 1:4) for each of the wheat groups. Amplification efficiencies based on slopes of standard curve showed that these primers had high efficiencies ranged from 98.7 to 107.0 %, except *FST* gene amplified in wild pool (84.7 %) and *6-SFT* gene amplified in cultivar pool (113.3 %). Meanwhile, the R^2^ values ranged from 0.994 to 0.999, except *CesA-2* amplified in landrace pool (0.794) (Additional file 7: Table S5). As shown in Fig. 6, the fold-changes in gene expression determined by QPCR were quite consistent with their normalized read counts (expression level) determined by RNA-seq. Namely, all nine genes selected for QRT-PCR validation exhibited a considerable reduction in their expression level from wild to domesticated groups.

## Discussion

Plant domestication has fascinated scientists interested in the evolutionary process ever since Darwin. Primary efforts were aimed to discover the wild progenitors of domesticated plants using classical taxonomy and genetics. Subsequently, phylogenetic distances between wild and domesticated plants were established by DNA markers including RFLP, SSR, AFLP, DArT and SNP [54]. Ayal et al. [25] were the first to address the questions related to wheat domestication by studying alterations in the transcriptome using cDNA microarray. They found 63 up- or down-regulated genes between wild and domesticated wheat. With the development of NGS technology, there was tremendous progress in the evolutionary studies aimed at unravelling the molecular basis of domestication using RNA-seq that can detect expression changes in thousands of genes. To the best of our knowledge, our study is the first that used RNA-seq to compare domesticated and wild tetraploid wheat glumes.

The transition from brittle rachis to non-brittle rachis was probably the first (pristine) domestication event. After the domestication episode, wheat glumes were subject to selection that made them more suitable for human needs. Some of the consequences were the emergence of easier to thresh spikes, which have a lower percentage of chaff, i.e., an increased proportion of the total kernel weight in the spike weight compared to the wild wheat. The wild and the landrace accessions of tetraploid wheat selected for this study have tough glumes and hulled seeds, which are non-free threshing. In contrast, the modern cultivars are free threshing (have soft glumes and non-hulled seeds). In our previous study related to threshing time, the three studied groups showed a pattern of gradual decrease, consistent with the chronological time frame from wild to landrace and from landraces to modern cultivars [16]. To some extent, the noted phenotypic difference could be caused by the observed lower expression level of genes related to the cell wall composition and glumes toughness (e.g., genes in the lignin biosynthesis pathway including *PAL*, *4CL* and *CCR*) in the domesticated genotypes. Furthermore, there was a significant increase in the SpHI in landraces compared to the wild wheat accessions and a slight improvement in the assayed modern cultivars compared to the landraces. This increase in the SpHI could be a consequence of the finer glumes and up-regulation of genes involved in the transport of amino acids (e.g., amino acid permease), which can facilitate in N retranslocation and grain filling [55].

We selected hulled-glume wild and landrace accessions for comparison with free-threshing modern cultivars, in order to search for DEGs that may be associated with evolution under domestication. Since the wheat genome is not completely sequenced yet, we used the wheat survey sequence [41] that provides the information needed for phasing homeologs of the A and B genomes. Until now, there is no reliable draft genome sequence in tetraploid wheat. However, the sequences of chromosome 5B, which is the first genomic survey sequence in wild emmer wheat, has been published recently [56] Our results detected 123,370 transcripts in the cultivar pools which are slightly lower than in the published *Triticum turgidum* transcriptome (140,118) built by the *de novo* assembly method [57]. The correspondence between the two studies is very good, despite the fact that we analysed only glumes at heading time while Krasileva et al. [57] analysed young roots, young shoots, spikes and grains. The possibility that there may be less expressed transcripts in glumes than in other organs is consistent with a previous RNA-seq study of different tissues in barley [55].

The identified DEGs may be sought as genes that were either preferred or rejected not by the early farmers and due to their association with traits were subject to selection efforts during improvement evolution under domestication. Yet, the possibility that some changes in expression patterns was a result of correlated responses to selection caused by tight linkage or linkage disequilibrium of corresponding genes with agriculturally beneficial alleles rather than directional selection should not be overlooked. Since there is a correlation between glumes shape and some agronomic traits [39], it could be speculated that at some time point(s) during evolution under domestication, the shape of glumes served as an indication/marker for the presence or absence of specific traits of interest.

### Candidate genes for wheat evolution under domestication

To understand changes in gene expression that occurred during evolution under domestication of tetraploid wheat, we selected 39 candidate DEGs in glumes for further characterization. Of these genes, 22 DEGs had lower expression in domesticated wheat; some are related to cell wall organization or biogenesis. In general, major components of plant cell wall are cellulose, hemicellulose, pectin, lignin and protein. However, we are not aware of other studies of genome expression in the glumes in the context of wheat domestication. Among the 22 down regulated DEGs we identified the following cell wall related genes: *CesA* genes are responsible for cellulose synthesis, and evolved in primary and secondary cell wall development of wheat [58]. *FLA*, a subset of arabinogalactan protein (AGP), has both an AGP-like glycosylated region and a putative fasciclin domain, which may contribute to cell adhesion, communication and cell wall architecture in *Arabidopsis*, rice and wheat [59, 60]. *TBL* is a protein family containing a plant-specific DUF231 domain and may be involved in biosynthesis and deposition of secondary wall cellulose in *Arabidopsis* [61]. Pectin is also one of the most important components of the primary cell wall in plants. We also found DEGs related to pectin metabolism, such as genes encoding pectin lyase-like protein and pectin acetylesterase family protein, which were down-regulated in domesticated compared to wild wheats. The lignin is considered as a major component of the secondary cell wall, providing the strength in plants. We have identified a series of DEGs in the pathway of lignin biosynthesis, including *PAL, CCR*, *FST* and *4CL*, which is in agreement with previous studies of cotton [32]. Likewise, two genes encoding for laccases (*LAC*), which may be involved in lignin polymerization [62], were also down-regulated in domesticated wheat. All these genes were down-regulated in the glume of domesticated wheat, suggesting that cell wall synthesis in glumes has undergone a kind of loss/reduction of function during evolution under domestication. In this study, we also observed that *CER1* (*eceriferum*) genes, which are associated with plant cuticular wax production [63], were significantly down-regulated in domesticated wheat. These findings are in agreement with higher wax content in the surface of glumes in wild tetraploid wheat genotypes [64].

In addition to the genes that are typically involved in cell wall composition, we identified a *COBRA* gene that was expressed only in the glumes of wild emmer wheat (i.e., was down-regulated in domesticated wheat). *COBRA* encodes for a plant-specific glycosylphosphatidylinositol (GPI)-anchored protein with ω-attachment site at the C terminus, a hydrophilic central region, a CCVS domain, a potential N-glycosylation site, N-terminal secretion signal sequence, and a predicted cellulose binding site. Extensive studies have demonstrated that *COBRA* is critical for biosynthesis of cell wall constituents comprising structural tissues of roots, stalks, leaves and other vegetative organs [65]. Likewise, it was suggested recently that genes from the COBRA family were involved in deposition of crystalline cellulose into different secondary cell wall structures [66].

Among the 22 down-regulated DEGs in the domesticated accessions we identified one transcription factor containing a NAC domain protein gene. NAC (NAM, ATAF1/2 and CUC2) domain proteins are plant-specific transcription factors known to play diverse roles in various plant developmental processes. The NAC domain gene, which was cloned from wild emmer wheat, accelerates senescence and could enhance nutrient remobilization to the developing kernels, thereby improving their nutritional content [67]. It is noteworthy that in barley, regulation of gene expression in glumes development may have direct connection with remobilization and accumulation of nitrogen in seeds, as was recently shown with respect to *HvAAP* genes [37, 55]. It was demonstrated that the shattering genes with a NAC domain, which functionally activates secondary wall biosynthesis and promotes the significant thickening of secondary walls by its high expression level, are present in *Arabidopsis*, rice and soybean genomes [68]. This suggests that NAC domain protein may be related to the control of the wheat shattering glumes and may have played a role in cereals and legumes domestication. According to our findings on DEGs down-regulated in the glumes of domesticated accessions compared to the wild progenitor, we can speculate that higher expression of cell wall controlling genes in wild wheat plays an important role in its glumes toughness.

Among the 17 DEGs that were up-regulated in glumes of domesticated wheat compared to the wild progenitor, we identified genes related to fatty acid elongation, flavonoid biosynthesis and amino acid transport. The most abundant up-regulated DEGs in domesticated wheat were *KCS* gene family. The *KCS* gene, a fatty acid elongase, determines fatty acid chain length and substrate specificity of the condensation reaction, a rate limiting step, and its subsequent elongated products like alkanes, aldehydes, primary alcohols, secondary alcohols, ketones and wax esters [69]. Another example of up-regulated genes in domesticated wheat was five *CHS* genes involved in the initial step of flavonoid biosynthesis, in the phenylpropaoid pathway, in pigments production, and plant resistance to biotic and abiotic stresses [70]. In addition, we found higher expression of a silicon transporter gene in the domesticated wheat which may be related to Si element uptake and distribution [71].

As mentioned above, regulation of *AAP* genes’ expression in barley glumes may play a role in nitrogen remobilization and accumulation in seeds [37, 55]. Based on the over-expression of *AAP* genes in glumes and increased SpHI in domesticated wheat compared to wild progenitor, we could speculate that dry matter allocation from the glumes to grain filling has increased during wheat evolution under domestication.

## Conclusions

In the current study we employed a comparative transcriptome profiling of wheat glumes in wild emmer, hulled landraces and modern cultivars. We have identified a few genes showing differential elevated expression levels at heading time that may be related to glumes toughness and could probably be involved in wheat evolution under domestication. Interestingly, we did not find any significant differentially expressed genes with AP2 domain similar to *Q* genes. It is considered that the wheat *Q* gene confers soft glumes and influences a series of traits involved in the control of domestication related traits such as brittle rachis, spike architecture and flowering time [14]. Likewise, we did not find differential expression in the *Tg* that confers glumes toughness. This fact may be considered as indirect evidence that these genes, start to elevate their expression level after heading time and culminate before ripening.

The advance in new genomic approaches provides new insight into domestication and evolution under domestication. It can facilitate the understanding of the origin of agriculture, mobilization of the adaptive potential of the wild and landrace germplasms, and finally, for the rethinking on breeding strategies for the accelerated improvement under domestication. Our results show that in addition to the classical domestication genes, there are many other genes differentially expressed between the wild genotypes, landraces and modern cultivars, which may be involved in control of agriculturally important traits and basic biological processes, plant development, cell wall composition, stress tolerance, and pigmentation. The major advantages of RNA-seq technology is that it can assist in unravelling candidate genomic/genetic targets of domestication and improvement selection even if nothing is known about the causal selected phenotype and it is not only limited to measurable phenotypic traits.

### Availability of supporting data

Raw reads of transcriptome have been deposited into the NCBI Short Read Archive (SRA, http://www.ncbi.nlm.nih.gov/sra/) under the accession numbers: SRR2084071, SRR2084163, SRR2084091, SRR2084165, SRR2084092, and SRR2084160.

## Additional files

Additional file 1: Figure S1. Pearson correlation between mapping ratios and genetic distance. (PDF 7 kb) Additional file 2: Table S1. DEGs in cultivar compared to wild wheat. (XLSX 246 kb) Additional file 3: Table S2. DEGs in landrace compared to wild wheat. (XLSX 184 kb) Additional file 4: Table S3. DEGs in domesticated compared to wild wheat. (XLSX 90 kb) Additional file 5: Figure S2. Gene Ontology classifications of novel transcripts. (PDF 97 kb) Additional file 6: Table S4. Differentially expressed transcripts. (XLSX 53 kb) Additional file 7: Table S5. Amplification efficiency of QRT-PCR primers. (DOCX 15 kb)
