# Supplementary figures and images for: Transcriptome profiling of wheat glumes in wild emmer, hulled landraces and modern cultivars
Source: BMC Genomics. 2015 Oct 13;16:777. doi: 10.1186/s12864-015-1996-0 (PMC4603339; doi:10.1186/s12864-015-1996-0)

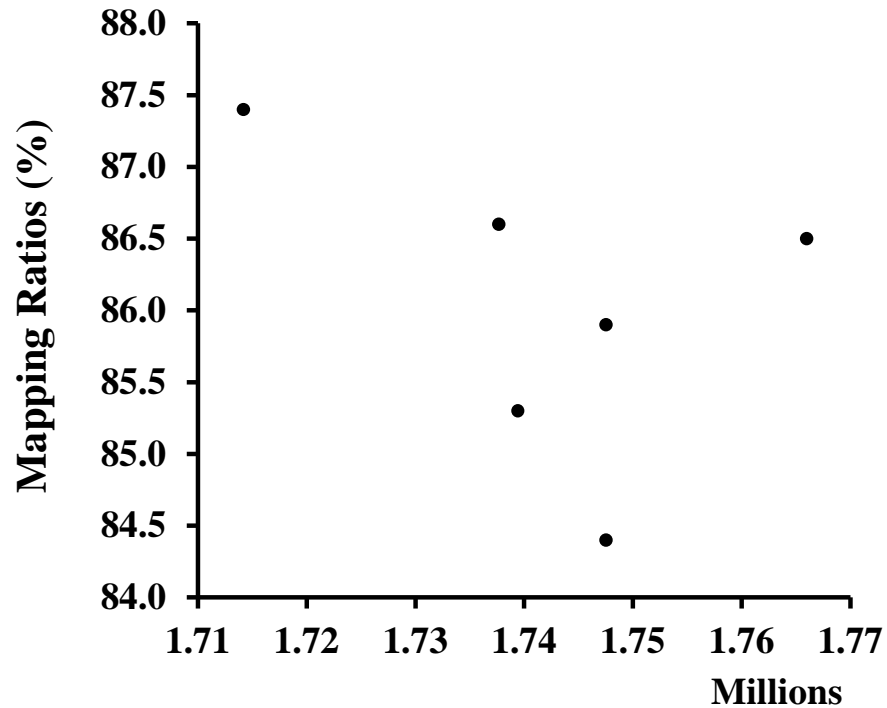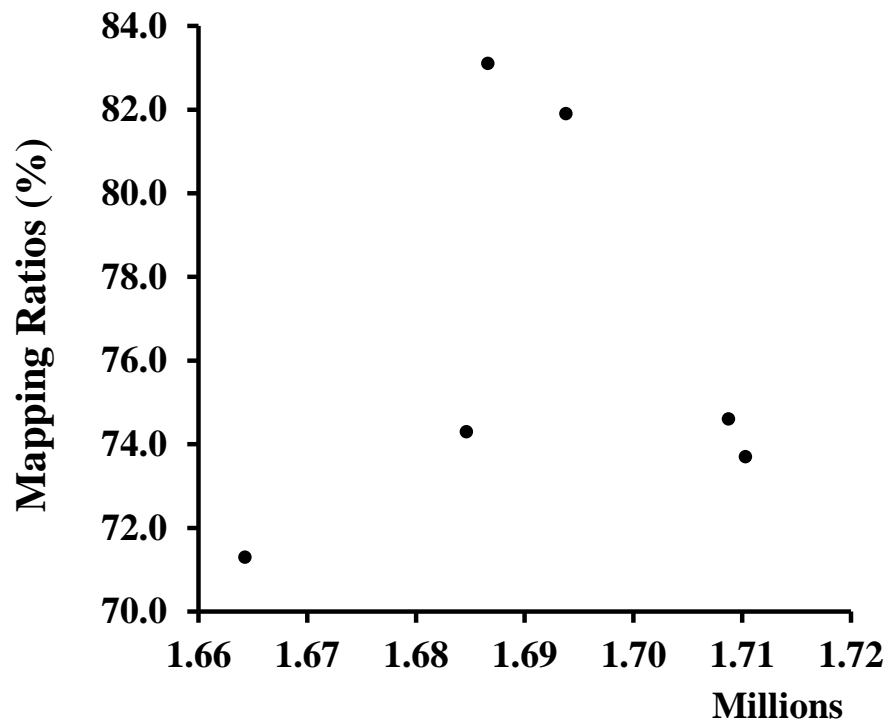

Supplement: Additional file 1: Figure S1. — Pearson correlation between mapping ratios and genetic distance. (PDF 7 kb) [file 12864_2015_1996_MOESM1_ESM.pdf]

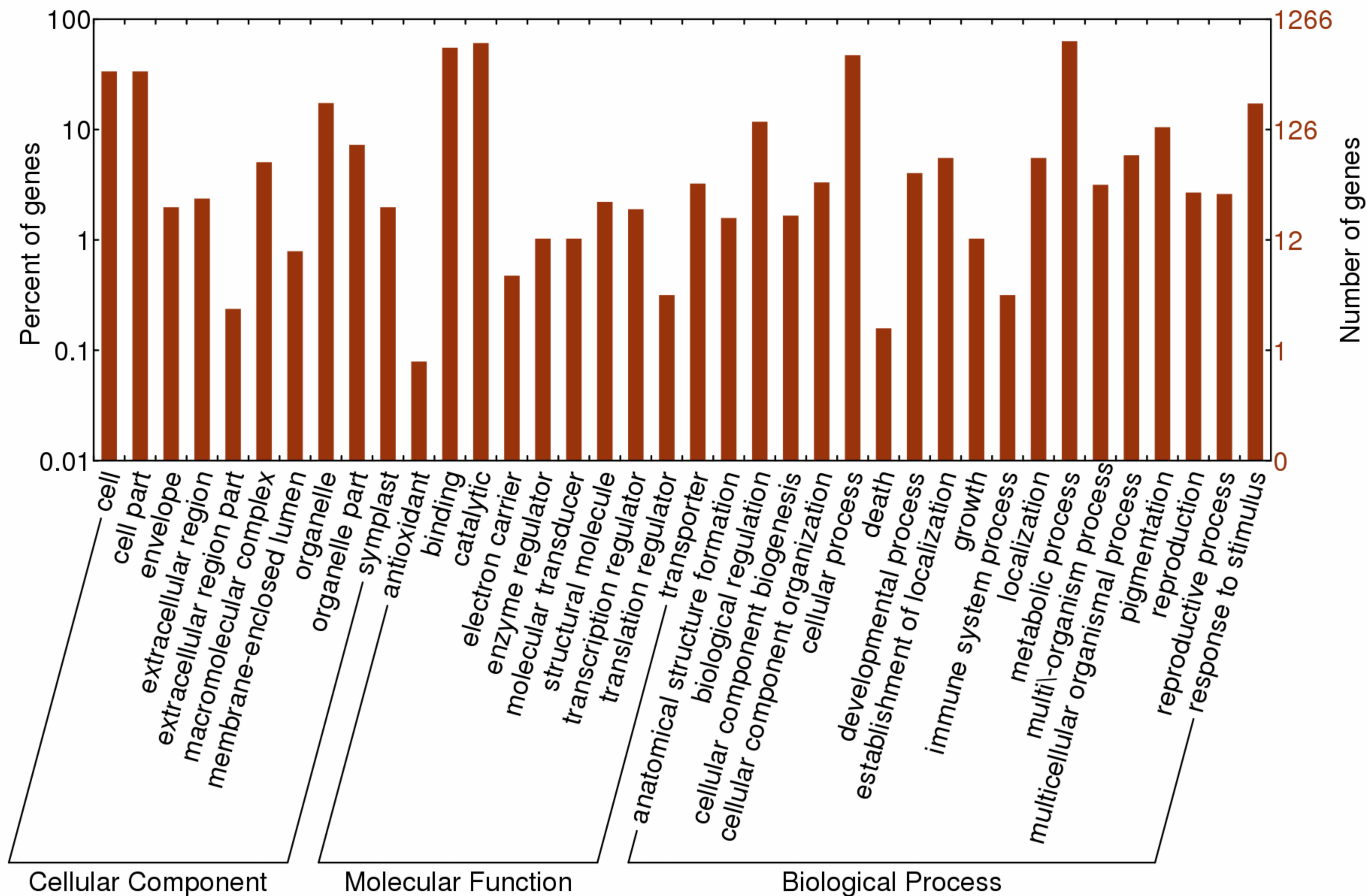

Supplement: Additional file 5: Figure S2. — Gene Ontology classifications of novel transcripts. (PDF 97 kb) [file 12864_2015_1996_MOESM5_ESM.pdf]
