# Supplementary material for: Transcriptome profiling of wheat glumes in wild emmer, hulled landraces and modern cultivars
Source: BMC Genomics. 2015 Oct 13;16:777. doi: 10.1186/s12864-015-1996-0 (PMC4603339; doi:10.1186/s12864-015-1996-0)
Supplement: Additional file 7: Table S5. — Amplification efficiency of QRT-PCR primers. (DOCX 15 kb) [file 12864_2015_1996_MOESM7_ESM.docx]

| **Gene** | **ID** | **Group** | **R^2^** | **Eff%** |
| --- | --- | --- | --- | --- |
|  | Ta3bLoc003710.1 | W | 0.999 | 102.018 |
| *FLA* |  | L | 0.997 | 102.546 |
|  |  | C | 0.995 | 104.075 |
| *FST* | Ta3bLoc056384.1 | W | 0.994 | 84.718 |
|  | Ta4asLoc013789.1 | W | 0.999 | 104.219 |
| *LAC16* |  | L | 0.998 | 107.452 |
|  |  | C | 0.995 | 98.675 |
|  | Ta4blLoc021918.2 | W | 0.995 | 100.039 |
| *LAC* |  | L | 0.999 | 106.275 |
|  |  | C | 0.996 | 98.671 |
|  | Ta5alLoc000723.1 | W | 0.998 | 100.682 |
| *CesA-2* |  | L | 0.794 | 106.779 |
|  |  | C | 0.994 | 98.817 |
|  | Ta6bsLoc005412.1 | W | 0.999 | 102.467 |
| *6-SFT* |  | L | 0.997 | 107.017 |
|  |  | C | 0.999 | 113.3 |
